# Elevated serum CA199 levels in patients suffering type 2 diabetes vs. various types of cancer

**DOI:** 10.1186/s12902-024-01539-y

**Published:** 2024-01-12

**Authors:** Yong Zhuang, Qingyan Cai, Xin Hu, Huibin Huang

**Affiliations:** Department of Endocrinology, The Second Affiliated Hospital of Fujian Medical University, No. 950 Donghai Street, Fengze District, Quanzhou City, Fujian Province 362000 China

**Keywords:** CA199, Tumour markers, Tumours, Type 2 diabetes

## Abstract

**Aims:**

Carbohydrate antigen 199 (CA199) is a standard tumor marker, and recent studies have found elevated in CA199 levels in patients with diabetes. However, there is no systematic measurement and comparison of serum CA199 levels in patients with diabetes and cancer. Here, a detailed description of the changes in serum CA199 levels in patients with type 2 diabetes and various cancers was explored.

**Methods:**

A total of 5,641 participants were screened for clinical laboratory test results of serum CA199 levels over the past three years (2020–2023). This study included 2,464 healthy controls, 688 patients with type 2 diabetes, and 2,489 patients with 16 different types of cancer. Each type of cancer had more than 30 independent serum CA199 level test results. The serum CA199 levels were compared between cancer groups, type 2 diabetes patients, and healthy controls. Additionally, the CA199 levels of cancer patients were compared with those of patients with type 2 diabetes.

**Results:**

The serum CA199 levels of esophagus cancer, lung cancer, pancreatic cancer, ovarian cancer, breast cancer, rectum cancer, prostate cancer, bladder cancer, liver cancer, gastric cancer, cervical cancer, colon cancer, lymphoma, thyroid cancer, intracranial tumors, and nasopharyngeal laryngeal cancer were found to be elevated compared to healthy controls (*P* < 0.01). In addition, the serum CA199 levels of patients with type 2 diabetes were also significantly elevated compared to healthy controls (*P* < 0.01). Moreover, the degree of elevation in serum CA199 levels in patients with type 2 diabetes was not significantly different from that observed in some types of cancer, such as esophagus cancer (*P* = 0.163), breast cancer (*P* = 0.927), prostate cancer (*P* = 1.000), bladder cancer (*P* = 0.406), Lymphoma (*P* = 0.975), thyroid cancer (*P* = 1.000), intracranial tumors (*P* = 0.161), nasopharyngeal and laryngeal cancer (*P* = 1.000).

**Conclusions:**

Serum CA199 levels also increase in type 2 diabetes, and the magnitude of the increase is similar to that seen in some cancers.

**Supplementary Information:**

The online version contains supplementary material available at 10.1186/s12902-024-01539-y.

## Introduction

Carbohydrate antigen 199 (CA199) is a type of mucin protein that serves as a tumor marker and is found on the glycolipids of cell membranes. It was named CA199 because it was identified and isolated through its binding affinity to the mouse monoclonal antibody 116NS19-9 [1]. CA199 is a member of the Lewis antigen family and a standard tumor marker [2]. The serum CA199 level is elevated in cancer patients, especially in digestive system tumors such as pancreatic, liver, colon, and rectal [3].

As is well known, the global prevalence of diabetes mellitus is increasing yearly, and by 2021, the total number of adults with diabetes worldwide is expected to reach 537 million cases [4]. Tumor markers are commonly used as a screening tool for tumors in diabetic patients during medical examinations. Recent research has shown a specific correlation between blood glucose levels and serum CA199 levels in diabetic patients [5]. A study [6] suggested long-term hyperglycemia in diabetic patients can cause hyaline degeneration and tissue necrosis in pancreatic cells. In the nuclear cells, partial glycoprotein components, such as CA199, can be released into the bloodstream in large quantities, resulting in a significant increase in blood CA199 levels.

There has been no systematic study or comparison of serum CA199 levels between cancer patients and individuals with type 2 diabetes. In this study, we evaluated the relationship between CA199 concentration in diabetic subjects and regular participants and various types of cancer.

## Methods

### Case collection

We collected laboratory data on serum CA199 levels from healthy participants, type 2 diabetes patients, and clinically diagnosed cancer patients in the clinical laboratory of the Second Affiliated Hospital of Fujian Medical University over the past three years (2020–2023). Participants using drugs known to impact CA199 were excluded from the study. Individuals diagnosed with tumors were excluded from the diabetes group during the enrollment process. Likewise, individuals with diabetes were excluded when enrolling participants for the tumors group. The tumor markers CA199 were measured by the Swiss Roche automatic biochemical immunoanalyzer and electrochemiluminescence method. There were more than 30 cases included for each type of cancer patient. Therefore, our study had 2,489 patients with 16 different types of cancer, 688 patients with type 2 diabetes, and 2,464 healthy controls.

### Statistical analysis

Statistical analysis was performed on most of the data using SPSS version 19.0 and GraphPad Prism version 8.0. The serum CA199 levels were expressed as mean (SD), median, and interquartile range. Due to the non-normal distribution of the data, a rank transformation was performed before conducting Dunnett’s t-test. A p-value of less than 0.05 was considered statistically significant.

### Institutional review board statement

The study was conducted according to the guidelines of the Declaration of Helsinki, and approved by the Ethics Committee of the Second Affiliated Hospital of Fujian Medical University (approval code 462; approval date 14.01.2020).

#### Informed consent statement

Informed consent was obtained from all subjects involved in the study.

## Results

**Fig. 1:**
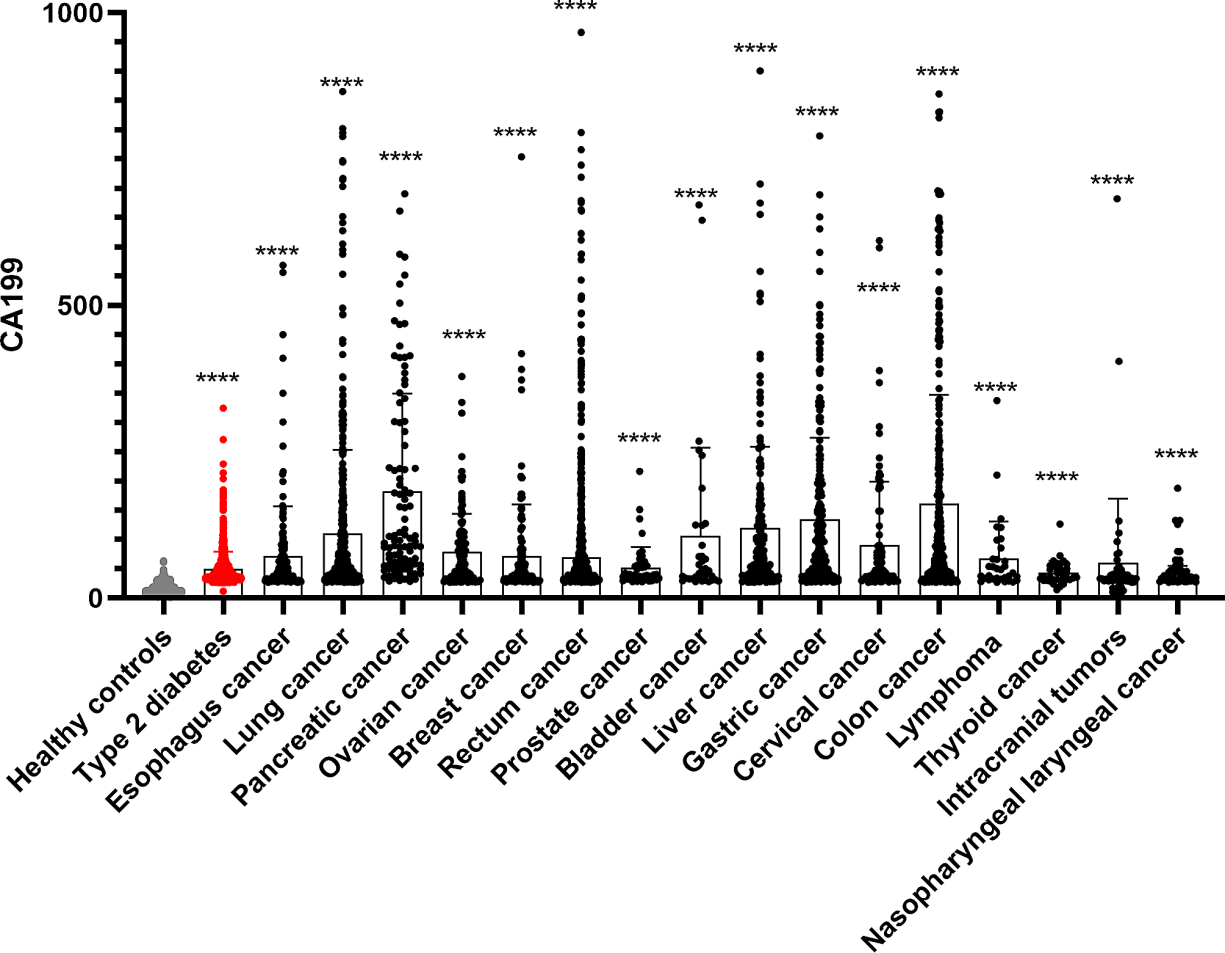
Comparison of serum CA199 levels in different groups

A total of 5,641 participants were screened for clinical laboratory test results of serum CA199 levels over the past three years (2020–2023). A total of 2464 healthy participants, 688 patients with type 2 diabetes, 162 esophagus cancer, 468 lung cancer, 102 pancreatic cancer, 118 ovarian cancer, 152 breast cancer, 419 rectum cancer, 48 prostate cancer, 36 bladder cancer, 189 liver cancer, 256 gastric cancer, 95 cervical cancer, 282 colon cancer, 33 lymphoma, 36 thyroid cancer, 47 intracranial tumors, and 47 nasopharyngeal laryngeal cancer were included. Based on the obtained data, we calculated and listed the mean, median, and interquartile range of serum CA199 levels in Table 1. In a comparison of 16 types of cancer, type 2 diabetes, and healthy controls, it was found that the serum CA199 levels of cancer (esophagus cancer, lung cancer, pancreatic cancer, ovarian cancer, breast cancer, rectum cancer, prostate cancer, bladder cancer, liver cancer, gastric cancer, cervical cancer, colon cancer, lymphoma, thyroid cancer, intracranial tumors, and nasopharyngeal laryngeal cancer) were higher than those of healthy controls (*P* < 0.05), and the CA199 levels of type 2 diabetes patients were also elevated compared to healthy controls (*P* < 0.05) (Table 1; Fig. 1). Compared with type 2 diabetes patients, it was found that the serum CA199 levels of lung cancer, pancreatic cancer, ovarian cancer, rectum cancer, liver cancer, gastric cancer, cervical cancer, and colon cancer were significantly higher (*P* < 0.01; *P* < 0.01; *P* < 0.01; *P* < 0.01; *P* < 0.01; *P* < 0.01; *P* = 0.004; *P* < 0.01); while there was no significant difference in the degree of elevation of serum CA199 levels between type 2 diabetes patients and patients with esophageal cancer (*P* = 0.163), breast cancer (*P* = 0.927), prostate cancer (*P* = 1.000), bladder cancer (*P* = 0.406), lymphoma (*P* = 0.975), thyroid cancer (*P* = 1.000), intracranial tumors (*P* = 0.161), and nasopharyngeal cancer (*P* = 1.000) (Table 2).

**Table 1:** The mean, median, and interquartile range of serum CA199 levels U/mL for healthy controls and patients with diabetes or other cancers

CA199	n	Mean (SD)	Median	InterQuartile Range	P value
**Healthy controls**	**2464**	**15.4(8.7)**	**13.0**	**12.1**	**1.00**
Type 2 diabetes	688	49.2(30.2)	38.9	21.7	< 0.01
Esophagus cancer	162	71.7(84.7)	42.6	38.7	< 0.01
Lung cancer	468	110.6(142.9)	53.6	74.1	< 0.01
Pancreatic cancer	102	182.6(167.1)	100.5	231.6	< 0.01
Ovarian cancer	118	78.8(65.3)	53.0	61.3	< 0.01
Breast cancer	152	71.2(88.3)	38.0	33.6	< 0.01
Rectum cancer	419	112.4(148.4)	51.9	67.7	< 0.01
Prostate cancer	48	51.7(35.6)	38.2	22.4	< 0.01
Bladder cancer	36	106.0(151.1)	44.3	83.1	< 0.01
Liver cancer	189	119.5(139.0)	64.4	96.2	< 0.01
Gastric cancer	256	134.3(139.9)	71.9	134.9	< 0.01
Cervical cancer	95	90.9(107.5)	46.5	53.2	< 0.01
Colon cancer	282	160.6(187.1)	74.4	164.9	< 0.01
Lymphoma	33	67.6(63.2)	42.6	43.7	< 0.01
Thyroid cancer	36	43.5(19.7)	37.9	23.1	< 0.01
Intracranial tumors	47	59.6(110.0)	37.6	39.0	< 0.01
Nasopharyngeal laryngeal cancer	46	50.0(33.3)	37.1	25.0	< 0.01

**Table 2:** Comparison of the rank of CA199 levels between various cancer groups and the type 2 diabetes group

Cancer groups vs Type 2 diabetes	P value
Esophagus cancer	0.163
Lung cancer	< 0.01
Pancreatic cancer	< 0.01
Ovarian cancer	< 0.01
Breast cancer	0.927
Rectum cancer	< 0.01
Prostate cancer	1.000
Bladder cancer	0.406
Liver cancer	< 0.01
Gastric cancer	< 0.01
Cervical cancer	0.004
Colon cancer	< 0.01
Lymphoma	0.975
Thyroid cancer	1.000
Intracranial tumors	0.161
Nasopharyngeal laryngeal cancer	1.000

## Discussion

Based on a study of serum CA199 levels in China regarding diabetes and cancer, our research revealed that not only did CA199 levels significantly increase in cancer patients and diabetes patients compared to healthy individuals. Compared to patients with diabetes, the serum CA199 levels in most cancer types (lung, pancreatic, ovarian, rectal, liver, gastric, cervical, and colon cancer) were significantly higher. In contrast, in some cancer types (esophageal cancer, breast cancer, prostate cancer, bladder cancer, lymphoma, thyroid cancer, intracranial tumor, and nasopharyngeal cancer), the degree of increase in serum CA199 levels was comparable to that in type 2 diabetes patients.

Diabetes is the fifth leading cause of death worldwide, and its incidence is increasing yearly [7]. The relationship between type 2 diabetes and cancer has been a subject of interest among many scholars [8]. Epidemiological data shows that the incidence of cancer among diabetes patients is also rising, especially for cancers such as pancreatic, liver, and breast cancer [9]. Research has found that patients with type 2 diabetes have a significantly higher incidence of endometrial and hepatobiliary pancreatic cancer than those without diabetes [10]. It is believed that cancer and diabetes have become the most common causes of death after cardiovascular diseases. In clinical practice, there is an increasing emphasis on cancer screening among patients with type 2 diabetes. However, preventing overdiagnosis in patients with type 2 diabetes who develop cancer remains challenging for healthcare professionals.

CA199 is synthesized by normal pancreatic and biliary duct cells and gastric, colonic, endometrial, and salivary epithelial cells. Serum CA199 is a commonly used serum marker for cancer evaluation, with advantages such as convenient detection, rapid results, and high reproducibility, and is widely used in clinical practice. Although CA199 has high sensitivity, its specificity is not strong. Serum CA199 levels are generally elevated in most cancer patients, especially those with digestive system cancers [11–14], consistent with our research findings. We discovered that CA199 levels were significantly higher in patients with esophageal cancer, lung cancer, pancreatic cancer, ovarian cancer, breast cancer, rectal cancer, prostate cancer, bladder cancer, liver cancer, gastric cancer, cervical cancer, colon cancer, lymphoma, thyroid cancer, intracranial tumors, and nasopharyngeal cancer, compared to healthy controls. Specifically, serum CA199 levels were particularly elevated in lung cancer, pancreatic cancer, ovarian cancer, rectal cancer, liver cancer, gastric cancer, cervical cancer, and colon cancer.

However, CA199 is not only elevated in cancer patients but also in non-cancer patients. Studies [15–17] have found that the serum levels of CA199 in patients with type 2 diabetes are higher than those in the healthy control group, and poor blood sugar control is an independent factor affecting CA199 levels. The results of this study were consistent with previous related studies. The results showed that the serum CA199 levels in patients with type 2 diabetes were significantly higher than those in normal controls, suggesting that CA199 may be abnormally elevated in patients with type 2 diabetes. Our study also showed that the magnitude of CA199 elevation in patients with type 2 diabetes was similar to that in some cancer patients, such as esophageal cancer, breast cancer, prostate cancer, bladder cancer, lymphoma, thyroid cancer, intracranial tumors, and nasopharyngeal carcinoma.

Here, we will attempt to analyze the potential mechanisms underlying the elevation of serum CA199 in patients with type 2 diabetes, although we did not perform such an analysis in our study. A study [18] suggested that the blood changes in pancreatic tissue of patients with type 2 diabetes were related to the inflammation process in the pancreas. Since CA199 is expressed in the exocrine part of the pancreas, it can serve as a sensitive indicator for screening exocrine pancreatic damage. Therefore, the inflammation of the islets and the destruction of islet cells caused by hyperglycemic toxicity may be one of the reasons for the elevation of CA199 levels. Some studies [19, 20] suggested that insulin may be associated with the increased activity of intestinal lactose transferase in the biosynthesis process of CA199. A study also indicated that elevated serum CA199 levels in patients with type 2 diabetes may be due to a prolonged half-life of CA199 [21].

## Conclusions

In summary, serum CA199 levels also increase in type 2 diabetes, and even the elevation of CA199 in patients with type 2 diabetes is comparable to that in some cancers. Additionally, clinical doctors can provide explanations for patients with elevated serum CA199 levels who have diabetes but not cancer and determine whether further cancer screening is necessary based on the index situation. When diabetic patients are found to have elevated CA199, determine whether there is a tumor-related manifestation, whether to improve the relevant tumor evaluation, and at the same time pay attention to the changes in the CA199 level, if it is caused by high blood glucose, when the blood glucose control can be found that the CA199 level will be reduced. However, our study also has certain limitations. Although many cases were included in this study, it was a retrospective study from a single center and only involved participants from China. Furthermore, patients with type 2 diabetes were not further stratified based on their blood sugar levels.

Therefore, further prospective and multi-ethnic comprehensive studies are needed. If future studies confirm our results, then CA199 can specifically differentiate between diabetes, and tumors, and can make better judgments for clinicians.

### Electronic supplementary material

Below is the link to the electronic supplementary material.

Supplementary Material 1

## Data Availability

The datasets used or analysed during the current study are available from the corresponding author on reasonable request.
