# Supplementary material for: Elevated serum CA199 levels in patients suffering type 2 diabetes vs. various types of cancer
Source: BMC Endocr Disord. 2024 Jan 12;24:9. doi: 10.1186/s12902-024-01539-y (PMC10785517; doi:10.1186/s12902-024-01539-y)
Supplement: Supplementary file 1 — Supplementary Material 1 [file 12902_2024_1539_MOESM1_ESM.doc]

Supplement Comparison of general data in each group

| Croups | Case (male/female) | Age (years) | HbA1c (%) | Duration (years) | Metastasis(%) |
| --- | --- | --- | --- | --- | --- |
| Healthy controls | 1239/1225 | 51.1±9.2 | 4.8±0.5 | — | — |
| Type 2 diabetes | 348/340 | 52.2±8.2 | 8.8±2.9 | 8.6±3.6 | — |
| Esophagus cancer | 83/79 | 53.1±9.7 | 5.1±0.6 | 1.2±2.1 | 35.6 |
| Lung cancer | 239/229 | 51.9±8.9 | 5.2±0.5 | 2.8±1.5 | 27.9 |
| Pancreatic cancer | 52/50 | 52.7±7.7 | 4.9±0.8 | 0.9±1.3 | 56.3 |
| Ovarian cancer | 64/54 | 51.6±8.2 | 5.4±0.7 | 1.9±2.1 | 23.6 |
| Breast cancer | 80/72 | 52.7±9.7 | 5.3±0.5 | 2.3±1.9 | 14.9 |
| Rectum cancer | 215/204 | 53.0±8.7 | 5.3±0.6 | 2.1±1.5 | 22.4 |
| Prostate cancer | 25/23 | 51.9±9.0 | 4.9±0.9 | 1.5±1.7 | 31.1 |
| Bladder cancer | 20/16 | 52.7±7.9 | 5.0±0.4 | 2.7±2.1 | 29.9 |
| Liver cancer | 98/91 | 53.1±9.5 | 5.8±0.6 | 1.5±0.9 | 49.5 |
| Gastric cancer | 132/124 | 51.7±8.8 | 4.9±0.8 | 1.8±1.1 | 23.6 |
| Cervical cancer | 50/45 | 52.3±7.4 | 4.8±0.9 | 2.4±2.1 | 19.8 |
| Colon cancer | 145/137 | 50.9±9.9 | 5.4±0.5 | 2.0±1.4 | 18.6 |
| Lymphoma | 18/15 | 52.5±7.2 | 4.9±0.8 | 0.9±0.5 | 44.7 |
| Thyroid cancer | 20/16 | 51.4±9.4 | 5.1±0.6 | 3.5±3.7 | 0.0 |
| Intracranial tumors | 25/22 | 52.0±9.3 | 4.7±0.8 | 1.4±2.1 | 21.1 |
| Nasopharyngeal laryngeal cancer | 25/21 | 51.9±8.4 | 5.3±0.4 | 1.6±1.9 | 25.5 |

Table 2 Comparison of the rank of CA199 levels between various cancer groups and the type 2 diabetes group

| Cancer groups vs Type 2 diabetes | *P* value |
| --- | --- |
| Esophagus cancer | 0.163 |
| Lung cancer | <0.01 |
| Pancreatic cancer | <0.01 |
| Ovarian cancer | <0.01 |
| Breast cancer | 0.927 |
| Rectum cancer | <0.01 |
| Prostate cancer | 1.000 |
| Bladder cancer | 0.406 |
| Liver cancer | <0.01 |
| Gastric cancer | <0.01 |
| Cervical cancer | 0.004 |
| Colon cancer | <0.01 |
| Lymphoma | 0.975 |
| Thyroid cancer | 1.000 |
| Intracranial tumors | 0.161 |
| Nasopharyngeal laryngeal cancer | 1.000 |

(This table shows a comparison between type 2 diabetes and various cancer diseases. Due to the non-normal distribution of the data, a rank transformation was performed before conducting Dunnett's t-test.)
